# Livedoid Vasculopathy with Severe Debilitating Neuropathy in a Prior Professional Athlete

**DOI:** 10.7759/cureus.57812

**Published:** 2024-04-08

**Authors:** Ian Del Valle, Devlin J Farr, Shannon Downie, Devin Broadwater, Peter W Barnes, Nam Nguyen, Jamison Hofer

**Affiliations:** 1 Internal Medicine, Keesler Medical Center, Biloxi, USA; 2 Internal Medicine, Ohio University Heritage College of Osteopathic Medicine, Dublin, USA; 3 Pathology, Keesler Medical Center, Biloxi, USA; 4 Dermatology, Keesler Medical Center, Biloxi, USA; 5 Rheumatology, Keesler Medical Center, Biloxi, USA; 6 Neurology, Keesler Medical Center, Biloxi, USA

**Keywords:** immuno-fluorescence, derm-rheum, pathology derm, military medicine, punch biopsy, coagulopathy, lower extremity ulcers, neuropathy, atrophie blanche, livedoid vasculopathy

## Abstract

Livedoid vasculopathy (LV) can be a challenging diagnosis with an interesting pathophysiology. LV is an uncommon diagnosis that can be easily mistaken for more common skin conditions, especially in a person of color who may be underrepresented in pathology images used in medical education. LV has an average of five years from initial presentation to diagnosis, possibly due to providers not having it on their differential for lower extremity ulcerations. Prolonged time to diagnosis can potentially lead to life-changing complications. We present a case of a former professional sprinter who became debilitated by neuropathy secondary to complications from LV. He was seen multiple times and had an extensive work-up exploring a broad differential including autoimmune etiologies, hypercoagulable disorders, neuropathies, and other vascular disorders before reaching the diagnosis. This case emphasizes the importance of early diagnosis and treatment with a multidisciplinary team to help prevent the progression of these symptoms. We break down an extensive work-up that involves a multidisciplinary team including dermatology, hematology, neurology, rheumatology, and vascular surgery. This case will also highlight examples of LV in a patient with a dark skin complexion, which can be challenging to find in current literature. We additionally show images that demonstrate many of the classic pathologic findings associated with LV and how those can help lead to the diagnosis along with detailed descriptions of those findings. Classic physical exam findings including atrophic blanche and lower extremity ulcerations are highlighted. We also review LV's history, diagnosis, and treatment to help readers achieve a better understanding of the disease.

## Introduction

Livedoid vasculopathy (LV) was first described in 1955 and was originally named livedo reticularis with summer ulcerations [1]. LV is a rare vasculopathy with an estimated incidence of one in 100,000 per year according to a 2013 review [2]. Multiple names have been used to describe LV, but the preferred terminology is livedoid vasculopathy, as it consists of focal vascular lesions not caused by vasculitis. LV characteristically affects the bilateral lower legs and feet due to increased thrombotic and decreased fibrinolytic activity [2]. The increased microthrombotic activity is believed to cause initial dermal purpuric papules and macules with retiform borders. These lesions can progress to stellate ulcerations and end-stage atrophic blanche, which are atrophic white stellate scars seen with LV. The thrombotic damage can ultimately create an active sensory-motor axonal polyneuropathy.

Ulcerations from LV can be extremely painful and disfiguring and significantly affect a patient's quality of life [3]. Earlier recognition and diagnosis could prevent some of these debilitating side effects. LV can be easily confused for venous stasis ulcerations or vasculitis, which require significantly different management. While there are no clear treatment guidelines due to the rarity of the disorder and a lack of randomized controlled trials, a combination of anticoagulants, anti-platelets, fibrinolytics, and various immunosuppressants have shown some efficacy [4]. Our case explores a patient with LV-associated neuropathy and reviews the pathophysiology of this disease.

## Case presentation

**Figure 1:**
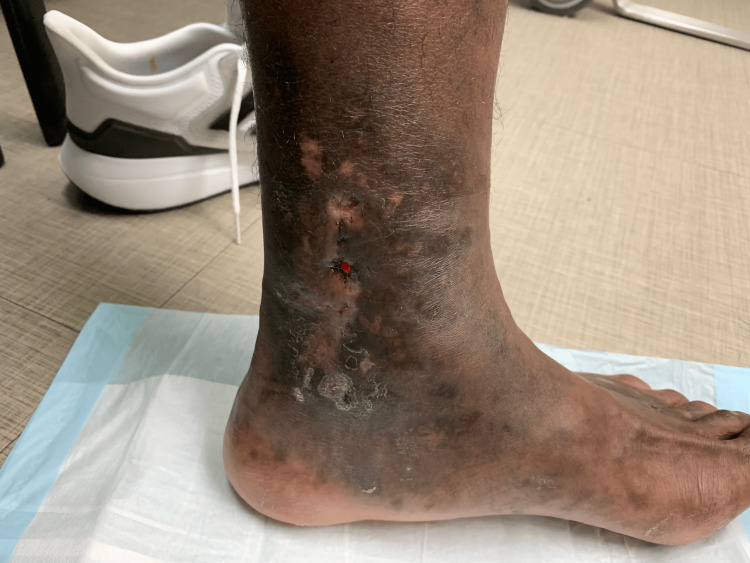
Punched-out ulcerations (1 cm) along the left dorsal foot and medial and lateral ankles.

**Figure 2:**
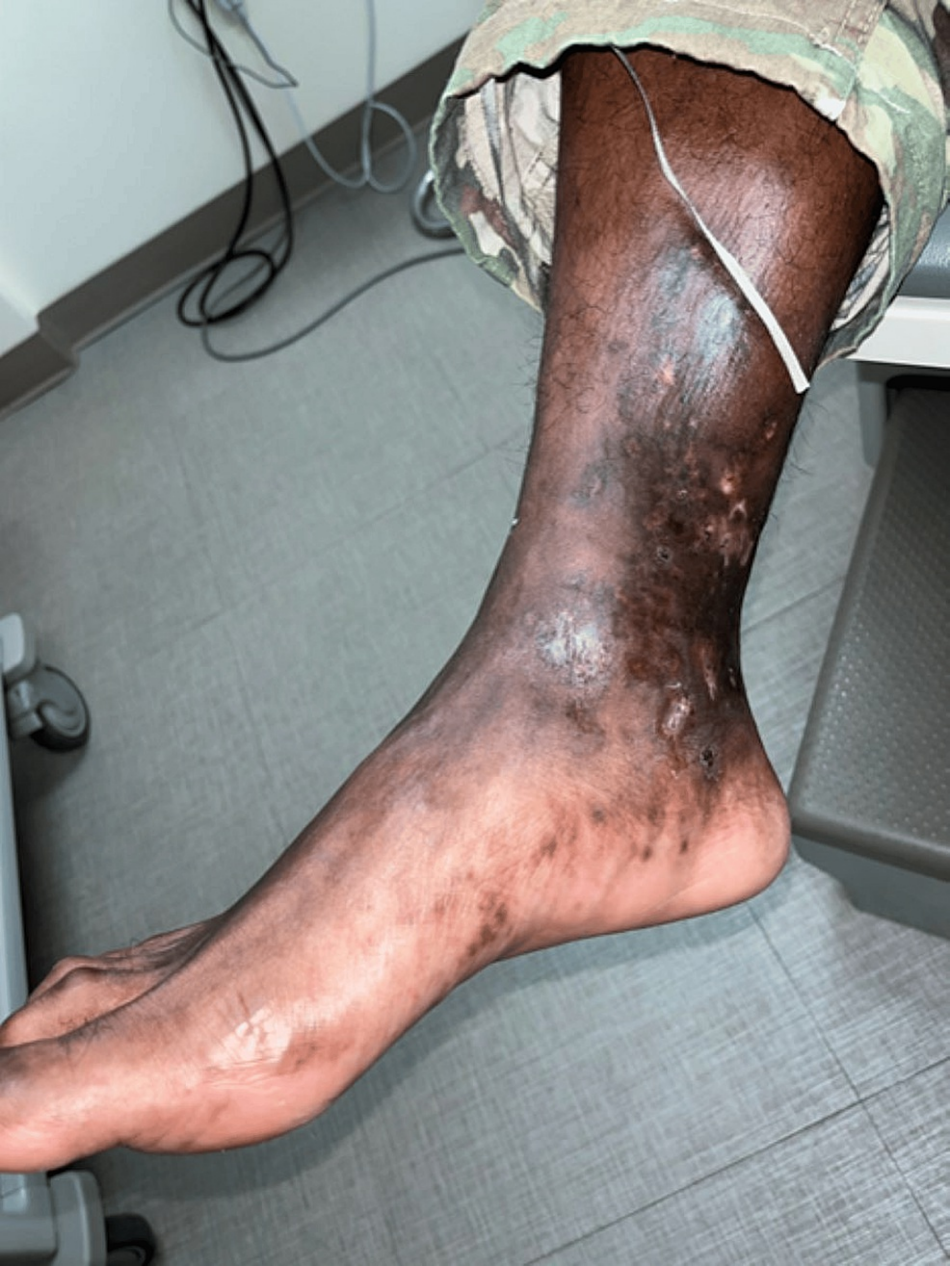
Atrophie blanche (atrophic white stellate scars) at the site of prior ulcerations on the right foot.

A 30-year-old Jamaican-American male presented to his primary care physician with the complaint of one month of bilateral foot pain and ulcerations with severe pain on the bottom of his left foot (Figures 1, 2). He was active-duty military and a former professional athlete with an unremarkable past medical history. He reported developing symptoms of numbness on the plantar surface of his bilateral feet. Numbness symptoms eventually progressed to pain, which was worse at night and did not improve with compression and over-the-counter (OTC) pain medications.

On physical exam, vibratory sensation throughout the lower extremities was intact; however, the patient had extreme tenderness to palpation on the left foot and decreased dorsiflexion and plantar flexion of both feet. Decreased sensation was noted on the plantar surface of his left foot.

Shortly after a trial of a regimen of compression stockings, ibuprofen, and acetaminophen, he presented to the emergency department with worsening foot pain. D-dimer was elevated and bilateral lower extremity ultrasound was negative for deep vein thrombosis (DVT). He was referred to a vascular clinic and was recommended to follow up with his primary care physician. At his vascular appointment, he had a normal ankle-brachial index of 0.96 on the right and 0.98 on the left. His primary care physician referred him to dermatology and neurology for further evaluation.

**Figure 3:**
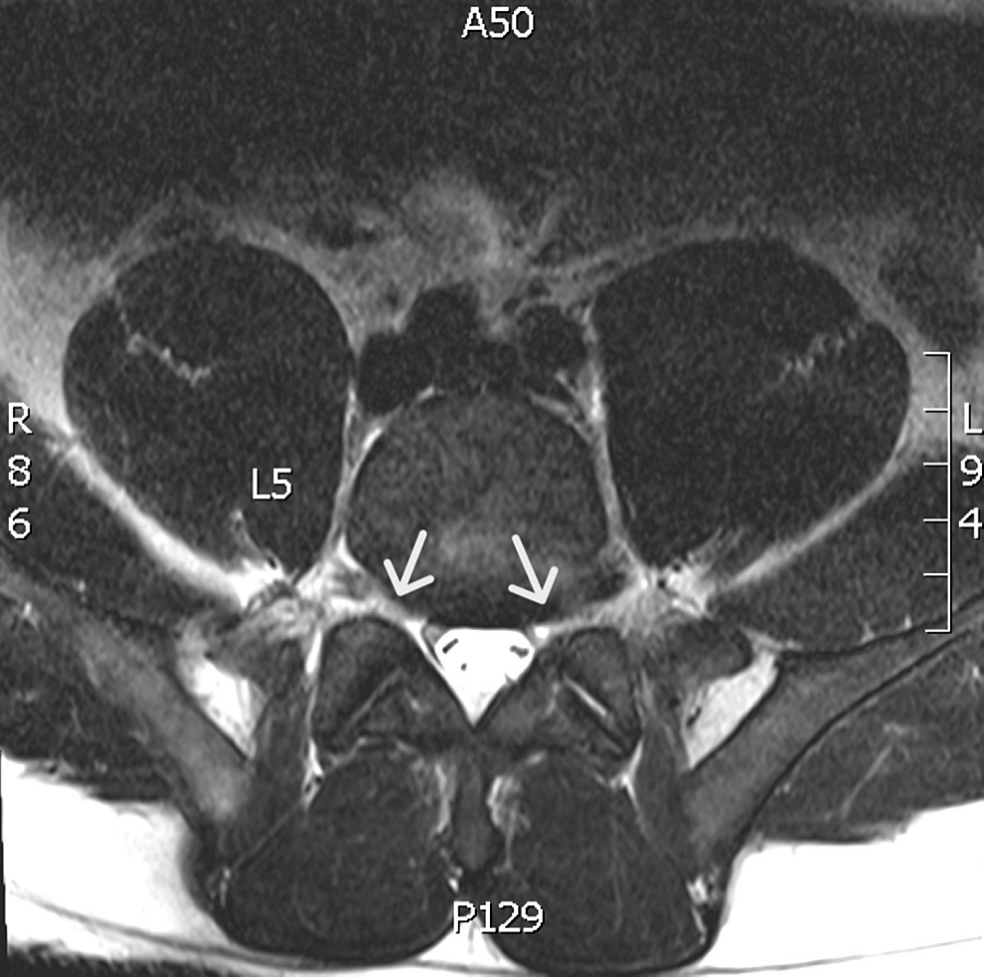
T2 weighted MRI demonstrating mild bilateral neural foraminal narrowing at L5-S1, shown with arrows.

He was evaluated by dermatology with a working diagnosis of lichen simplex chronicus and started on a topical steroid cream. At the patient’s neurology evaluation, his pain was noted to have significantly worsened. He now required a wheelchair as he was unable to ambulate. The patient had no improvement in pain or skin lesions despite using the prescribed steroid cream and analgesic therapy. Electromyography (EMG) was performed, which showed severe diffuse axonal sensorimotor polyneuropathy affecting the bilateral lower extremities. A magnetic resonance imaging (MRI) was ordered due to concern for L5 radiculopathy, which showed mild degenerative disc disease with mild bilateral neural foraminal narrowing at L5-S1 and no significant spinal canal narrowing (Figure 3).

Neurology referred the patient to rheumatology, hematology, and a second dermatologist for a second opinion. Rheumatology and hematology ordered a work-up for infectious and autoimmune etiologies. Laboratory evaluation showed C3 and C4 complement levels elevated to 177 mg/dL (82-167 mg/dL) and 51 mg/dL (12-38 mg/dL), respectively. Complement CH50 was also elevated at >60 U/mL (>41 U/mL). Anti-nuclear antibody test showed a positive 1:160 ratio in a speckled pattern. Cardiolipin antibody IgM was also elevated at 22 MPL/mL (0-12 MPL/mL). His remaining work-up was negative, including HIV, hepatitis A/B/C panel, anti-neutrophil cytoplasmic antibody (ANCA) panel, serum protein electrophoresis, kappa and lambda free light chains panel, and serum electrolytes.

**Figure 4:**
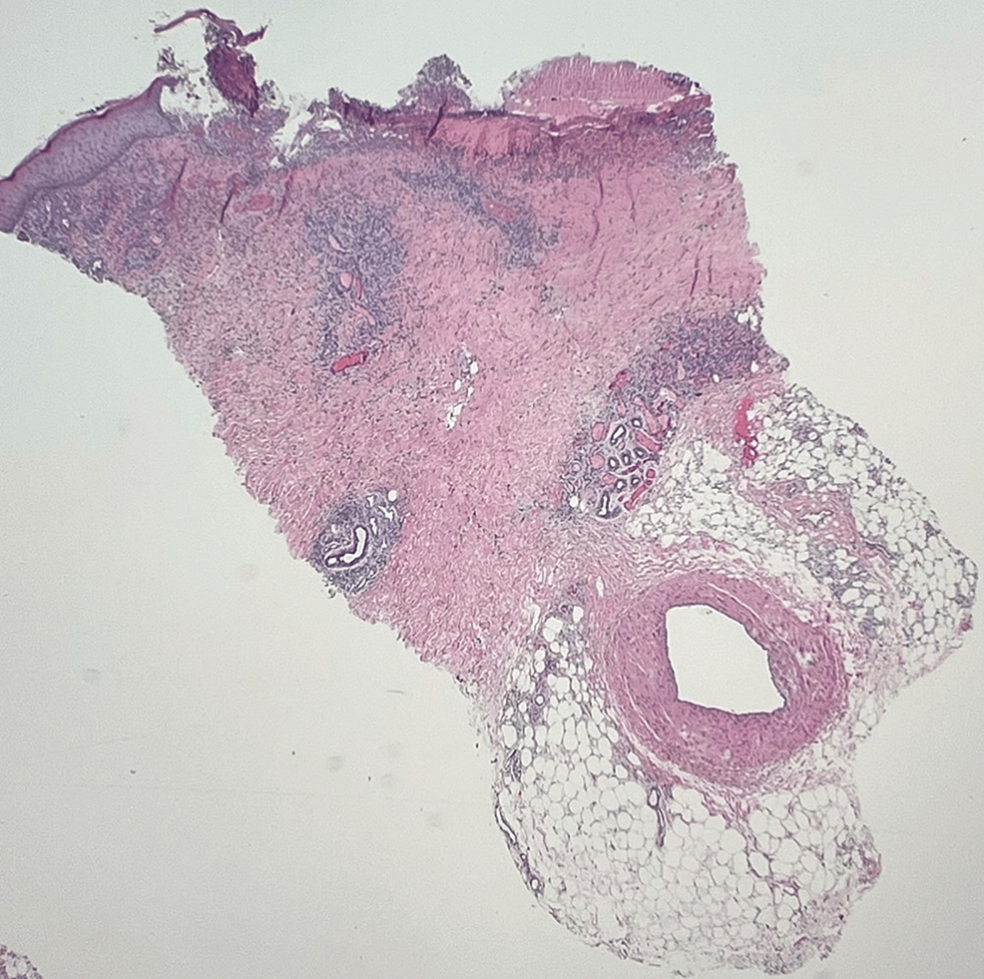
Photomicrograph (20x) of skin punch from left lower leg. A large caliber vessel is seen present in the deep subcutaneous without vasculitis changes.

**Figure 5:**
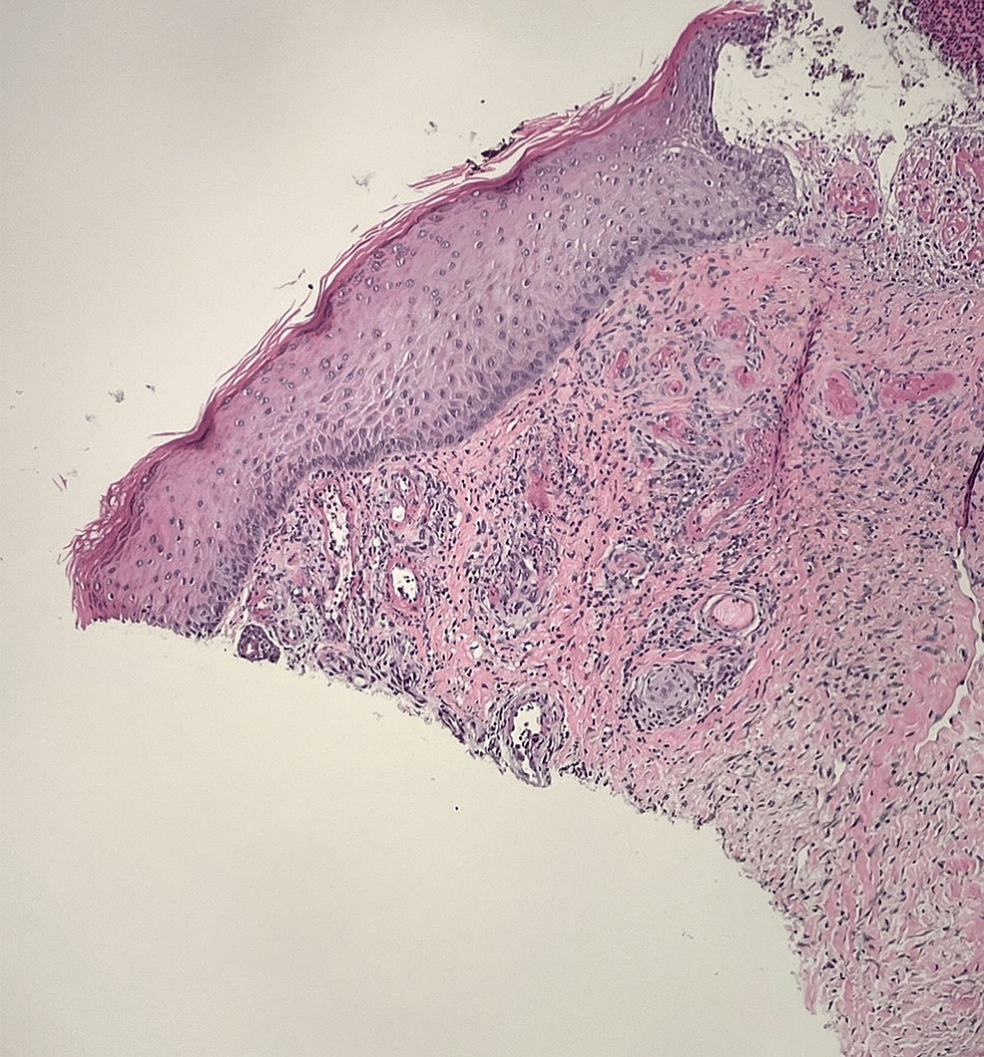
Photomicrograph (100x) of skin punch from left lower leg away from ulcer. Low power demonstrates perivascular inflammation with evidence of inflammatory cells within the vessel walls. There is intravascular thrombosis and eosinophilic fibrinous exudate within the vessel walls.

On the second dermatology evaluation, the patient was noted to have many punched-out ulcerations with stellate white scars. Given the concern for vasculopathy or vasculitis, punch biopsies for hematoxylin and eosin (H&E) and direct immunofluorescence (DIF) were performed. Initial microscopic review pathology found ulcerated epidermis with neutrophilic abscess, small vessels with fibroid necrosis of the vessel wall, and pigment-laden macrophages concerning for LV (Figure 4, 5). A Grocott methenamine silver (GMS) and periodic acid-Schiff (PAS) special stain were performed without evidence of a fungal infection. Given the severity of the findings, the reviewing pathologist elicited an opinion from a dermatopathology expert for further review.

Perivascular inflammation is seen with evidence of inflammatory cells within the vessel walls. There is intravascular thrombosis and eosinophilic fibrinous exudate within the vessel walls. The overlying, nonulcerated, epidermis is relatively unremarkable and uninvolved.

**Figure 6:**
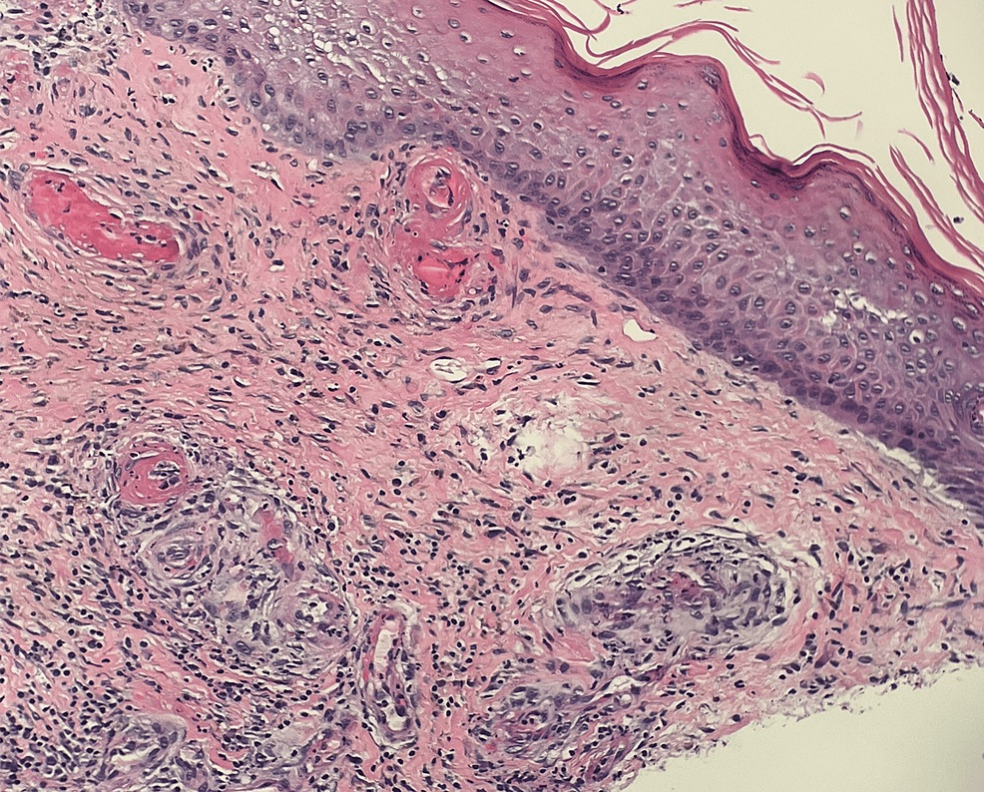
High power photomicrograph (200x) of skin punch from left lower leg away from ulcer, opposite bisected portion of skin punch, demonstrates similar morphologic findings as other half of biopsy.

Further evaluation by the dermatopathologist added that the vascular changes in the immediate vicinity of the ulcer bed are typically difficult to interpret, as they may be reactive. The vessels away from the ulcer demonstrated intravascular thrombosis with some eosinophilic exudate within the vascular walls. There was neovascularization noted. PAS, with and without diastase, was performed, demonstrating no intravascular hyaline deposits. Extensive hemosiderin was noted on the iron stain. CD31 and ERG immunohistochemistry highlighted vascular endothelium. The dermatopathologist stated the features are consistent with LV but recommended excluding lupus-like anticoagulant, protein C or S deficiency, or other hypercoagulable disorders. Figure 6 highlights the histopathology features demonstrated on histomicroscopy. An additional punch biopsy was sent for DIF. Immunofluorescence showed that there was IgM in the superficial, mid-dermal, and deep dermal blood vessels. C3 was 2+ clumps in the superficial, mid-dermal, and deep dermal blood vessels. Fibrinogen was 2+ in the superficial walls, mid-dermal, and deep dermal blood vessels, IgG, IgG4, and IgA were all negative.

The ulcers were determined to be characteristic of LV, and histopathology findings supported this diagnosis. Additional laboratory testing for underlying rheumatologic disorder or thrombophilia, including antiphospholipid antibodies, lupus anticoagulant, prothrombin mutation, protein C or S deficiency, and antithrombin deficiency, were without any significant findings or alternative diagnoses.

After diagnosis, the patient was initiated on aspirin 81 mg daily, pentoxifylline 400 mg three times daily, and lidocaine patches as needed for neuropathic pain. At his follow-up a few months later, a slow but steady improvement was noted in the patient's symptoms.

## Discussion

This case represents a common problem with patients presenting with LV: prolonged time to diagnosis due to the challenge in recognizing this disease process. Particularly notable in this case is that a former professional athlete and physically fit active-duty military member became debilitated to the point of requiring a wheelchair for mobility, highlighting the potential severity of LV-associated complications. LV is a particularly rare disease with an incidence of around one in 100,000, with females being affected three to one when compared to males [5]. Given its low incidence and sharing of symptoms with many more common pathologies, the time to diagnosis is estimated to be around five years from the initial presentation [3]. The differential diagnosis is broad and includes chronic venous insufficiency, peripheral vascular disease, vasculitis, pyoderma gangrenosum, skin trauma, neuropathy, infection, thromboangiitis obliterans, malignancy, microvascular occlusion disorders, and sickle cell disease. Understanding the pathogenesis and the factors contributing to delay in diagnosis could lead to earlier diagnosis in future cases.

Patients typically present with purpuric plaques and coalescing ulcerations on the lower extremities. In later stages, patients often present with atrophie blanche: white, stellate, smooth plaques, and scars with telangiectasias interspersed [5]. Due to the damage in the veins, a secondary stasis dermatitis can commonly occur, which obscures and can make the underlying diagnosis even more difficult.

While sometimes discussed as vasculitis, an important distinction has been made that the disease process may result in some inflammation that does not appear to be driven by inflammation like vasculitis. A case series comparing LV and cutaneous small vessel vasculitis (CSVV) showed that LV had significant platelet and lymphocyte activation, whereas CSVV showed high serum levels of proinflammatory cytokines, suggesting an important difference in pathophysiology [6]. The microvascular thrombosis found in LV is believed to be due to dysregulation in fibrinolysis; however, given the rarity of the disorder, the exact mechanism of the dermal venous thrombosis and subsequent skin changes is difficult to ascertain.

Diagnosis is achieved in correlation with clinical history and presentation. Biopsies typically show dermal neovascularization with intraluminal thrombosis, hyaline degeneration, and, more importantly, no lymphocytic vessel wall necrosis and immunofluorescence without immune complex deposition [7]. For definitive diagnosis, excluding vasculitis (ANCA, rheumatoid factor, etc.) and other hypercoagulable causes (factor V Leiden, protein C/S deficiency, etc.) is required. However, LV has also been described as occurring simultaneously with systemic lupus erythematosus (SLE), which would generally have a positive anti-nuclear antibody test, making the diagnosis a multidisciplinary challenge where the pathology may not align fully with laboratory testing [7]. In this particular case, although the patient had a positive ANA of 1:160, his clinical symptoms and other laboratory work-up were not consistent with SLE. Confounding the diagnosis further, histopathology of the ulcerations can vary depending on the age of the lesion. New ulcerations have small vessel changes, whereas chronic wounds show edema and neovascularization [3].

Neuropathy is not always a part of the presentation for LV. The young male, whose case was presented in this report, had neuropathy as the main complaint that eventually led to an LV diagnosis. The most likely pathologic mechanism of his neuropathy is suspected to be due to vaso-occlusion and subsequent ischemia of the vasa nervorum of the peripheral nerve [8]. One retrospective cohort study found the rate of peripheral neuropathy to be 12.73% and that these symptoms began on average 36 months after the cutaneous manifestations originally presented [9]. Interestingly, our patient presented with severe peripheral neuropathy despite noticing skin changes only one month prior.

Contributing to the prolonged time to diagnosis of this disease could be the paucity of examples of LV in darker skin tones found in the literature. In our literature review, we found very few photographic examples of this disease process in patients with darker skin complexion. We know from previous literature that skin conditions like melanoma are known to present at later stages in the Black and Hispanic populations [10]. Dermatologic conditions can present differently in different skin tones, and this needs better representation in medical training [11]. Adding images and discussions to clinical education that represent skin of color has been shown to improve student comfort when identifying these conditions [12]. One study found that many dermatology residents currently feel that their training to identify pathology in darker skin tones is inadequate [13]. Research is being done to create a more racially diverse database of dermatologic pathology to innovate the current curriculum for those studying medicine, and the images shown could be included in something similar [14].

Treatment of this condition is challenging, given that there are no guidelines due to low incidence. First-line treatment is typically a combination of antiplatelets and/or anticoagulants, along with compressive therapy and pain control [4]. Our patient was initiated on aspirin and pentoxifylline with slow improvement.

## Conclusions

We hope with this case that readers will be more equipped to recognize the distinct characteristics of LV earlier and initiate treatment in a timely fashion. LV should be considered in individuals presenting with painful lower extremity ulcers. Prolonged time to diagnosis, due to lack of familiarity with the disease, is common with LV and may lead to complications such as neuropathy. Our patient went from being a highly athletic runner to being wheelchair-bound from the original time of presentation to the time of diagnosis, which demonstrates the importance of prompt diagnosis. With earlier recognition and treatment, the progression of morbidity associated with severe pain and disability could be improved. Additionally, recognizing the presentation of LV in skin of color may be challenging due to difficulties in finding examples in the literature. This could be prevented by including images like the ones presented in the article in educational materials for healthcare providers. 
